# Long-term disability after neonatal encephalopathy in low-resource settings

**DOI:** 10.1038/s41390-025-04758-8

**Published:** 2026-01-13

**Authors:** Shona Goldsmith, Eleanor R. Gunn, Alistair J. Gunn, Nadia Badawi

**Affiliations:** 1 Cerebral Palsy Alliance Research Institute, The University of Sydney, Sydney, Australia; 2 Kidz First Neonatal Unit, Middlemore Hospital, Auckland, New Zealand; 3 Department of Physiology, The University of Auckland, Auckland, New Zealand; 4 Grace Centre for Newborn Intensive Care, Children’s Hospital at Westmead, Sydney, NSW Australia

Neonatal encephalopathy (NE), a “clinical syndrome of disturbed neurologic function in the first week after birth in an infant born at or beyond 35 weeks of gestation, manifested by an abnormal level of consciousness or seizures, often accompanied by difficulty with initiating and maintaining respiration and depression of tone and reflexes.”^1^ is a major global burden, with an estimated 1.2 million cases each year. Infants with NE have high risk of mortality with serious morbidity in survivors, particularly for those born in low- and middle-income countries (LMICs). It is therefore important to know whether risks are improving… or not.

The Global Burden of Disease (GBD) 2021 database offers an overarching estimate of global impact, as well as regional comparisons by socio-demographic index (SDI), GBD region, and 204 individual countries and territories. Using this database, Zhao and colleagues report a mixed “score-card” for NE across the globe.^2^ The bad news is that the prevalence of NE globally increased by ~14% between 1991 and 2021, with the largest increase in South Asia. Disparity by income level was clear: prevalence rates increased in almost all SDI regions, with the greatest rise in the low-middle and low regions, compared with a decline in high SDI regions.

More positively, among infants with NE, Zhao et al. found an overall fall in early mortality. Again, neonatal mortality was lowest and declined by an encouraging 63% in high SDI regions. For survivors of NE, they report an overall decline in disability-adjusted life years (DALYs) in all regions. Of considerable concern, however, in low SDI countries, there was a 21% increase in DALYs. The improved survival of high-risk infants in high SDI regions will presumptively reflect neonatal intensive care, widespread vaccination avoiding infectious mortality, and other public health strategies. While there is a risk of misinterpretation when comparing data sources from multiple, disparate countries, particularly given the lack of disability surveillance mechanisms in many LMICs, emerging data suggest a true increase in disability globally.^3,4^ The next step must be to dedicate time and effort towards reducing disability in LMICs.^5–7^

Importantly, Zhao et al. affirm that NE encompasses encephalopathies caused by a range of etiologies—both hypoxic ischemic (HIE) and others. There is considerable confusion over the term NE versus HIE, and they are often used interchangeably. By definition, HIE is a subset of NE.^8^ NE in general and HIE in particular may be associated with maternal risk factors, and adverse events from the preconception period, through early and late pregnancy, including genetic factors, socioeconomic vulnerability, congenital infection, metabolic disorders, stroke, congenital anomalies, abnormal placentas, and abnormal fetal growth.^9–11^

Both to support clinical decision making and to ensure appropriate comparison and pooling of research data, clear definitions and diagnostic criteria for NE are essential. An international network is currently working toward a consensus-based definition of NE and criteria for diagnosis (The DEFINE project), informed both by the literature and by a real-time Delphi approach including a broad range of stakeholders including healthcare providers, researchers, and people with lived experience of NE.^9^

The real-world importance of accurate assessment and diagnosis of NE has been highlighted by the very different outcomes of randomized controlled trials of therapeutic hypothermia (TH), a targeted therapy for HIE. In high income countries, TH was consistently associated with improved survival without disability.^12^ By contrast, the large Hypothermia for Moderate or Severe Neonatal Encephalopathy in Low-Income and Middle-Income Countries (HELIX) trial,^13^ found no effect on the risk of death or disability, and an apparent increase in the risk of death, even in centers with state-of-the-art tertiary neonatal care facilities. This important randomized controlled trial suggests that the underlying causes of NE are different in LMIC with a higher rate of MRI changes consistent with subacute, evolving fetal hypoxemia rather than acute hypoxia, in the context of fewer acute sentinel events and lower birth weights.^14,15^ In turn, this is highly consistent with previous evidence from the GBD database that low birth weight is a major factor underlying the high burden of neonatal mortality and age-standardized disability in low and low-middle SDI countries.^4^ These data strongly suggest that the increasing risk of disability in low SDI regions described by Zhao et al. will not be effectively addressed by transferring therapies from high-income countries to LMIC. Thus, further research in LMIC is vital to preventing NE as well as improving its consequent long-term outcomes.
